# Quasicrystals

**DOI:** 10.6028/jres.106.049

**Published:** 2001-12-01

**Authors:** John W. Cahn

**Affiliations:** National Institute of Standards and Technology, Gaithersburg, MD 20899-8555

**Keywords:** aperiodic crystals, new branch of crystallography, quasicrystals

## Abstract

The discretely diffracting aperiodic crystals termed quasicrystals, discovered at NBS in the early 1980s, have led to much interdisciplinary activity involving mainly materials science, physics, mathematics, and crystallography. It led to a new understanding of how atoms can arrange themselves, the role of periodicity in nature, and has created a new branch of crystallography.

## 1. Introduction

The discovery of quasicrystals at NBS in the early 1980s was a surprise [1]. By rapid solidification we had made a solid that was discretely diffracting like a periodic crystal, but with icosahedral symmetry. It had long been known that icosahedral symmetry is not allowed for a periodic object [2].

Periodic solids give discrete diffraction, but we did not know then that certain kinds of aperiodic objects can also give discrete diffraction; these objects conform to a mathematical concept called almost-^1^ or quasi-periodicity [3]. By definition all quasi-periodic objects diffract discretely, even though they are not periodic. Quasiperiodic objects can have any of the infinite set of point group symmetries listed as non-crystallographic in the International Tables for Crystallography [4]; because they have a single rotation axis of order 5, or one greater than or equal to 7, or have icosahedral symmetry with its six intersecting 5-fold axes.

Crystal periodicity has been an enormously important concept in the development of crystallography. Haüy’s hypothesis that crystals were periodic structures led to great advances in mathematical and experimental crystallography in the 19th century. The foundation of crystallography in the early nineteenth century was based on the restrictions that periodicity imposes. Periodic structures in two or three dimensions can only have 1,2,3,4, and 6 fold symmetry axes. With no exceptions, each crystal was found to conform to one of only 32 ways of combining these symmetry axes, the so-called “crystallographic” point group symmetries. External forms of periodic crystals were found to be limited to combinations of only 47 forms (32 general and 15 special) made of symmetrically arranged bounding planes [5]. Cubes, octahedra, and tetrahedra, for instance, are examples of special forms belonging to the cubic point groups, octahedra to point groups 432, $m\bar{3}$, and $m\bar{3}m$, tetrahedra to 23 and $\bar{4}3m$, and cubes to all five. In the nineteenth century each known crystal could be fit into one (or more) of these 32 point groups by the examination of its external form. That no additional form was found could be taken as proof that all crystals are periodic. Regular icosahedra and dodecahedra are special forms of both icosahedral point groups, 235 and $m\bar{3}\bar{5}$. All icosahedral forms have fifteen intersecting 2-and ten intersecting 3-fold axes, as well as six intersecting non-crystallographic 5-fold axes.

With the assumption of periodicity, the mathematical aspects of crystallography were set and completely worked out in the 19th century; that aspect became an almost closed field. In two and three dimensions the number of crystal systems, point groups, and plane or space groups were all enumerated. When the allowed symmetry axes are combined with translations, it was shown that there are only 230 space groups in three dimensions. In two dimensions there are only ten point groups and seventeen plane groups. An elementary proof why this listing contains every case allowed by periodicity and why no others are allowed has long been available in popular mathematics books [6]. Such complete listing are called catalogs. Each one of the seventeen are beautifully illustrated in etchings by M. Escher [7], as well as Moorish tilings and Turkish carpets. Extensions were developed for color groups and for crystallography in higher dimensions. Magnetic structures and their 1609 Shubnikov space groups are an example of such an extension in which spins, up or down (or two colors), are treated as if in a fourth dimension [8].

With the advent of x-ray diffraction in 1912, external form became less important. Crystals became defined as periodic arrangements of identical unit cells. The dominant work of crystallographers became structure determinations by diffraction to find the atom content of one unit cell. The method depends on an assumed periodicity, and the results usually confirmed it.

## 2. Discussion

Had we found a crystal? Many definitions of crystals are in use, some have changed over the centuries. Our solid was metallic and thus not a “clear transparent mineral.” It can be grown to form “convex solids enclosed by symmetrically arranged plane surfaces, intersecting at definite and characteristic angles.” According to the latter of these older definitions, quasicrystals are crystals. The discovery in 1912 that crystals could diffract x-rays discretely implied either their periodicity or quasiperiodicity. But as noted above, the subsequent structure determinations, seem to have led to the acceptance of a definition of crystals based on the periodicity of their internal structure, and one which unnecessarily ruled out quasiperiodicity. But by 1992 the IUCr Ad Interim Commission on Aperiodic Crystals wrote “by ‘crystal’ we mean any solid having an essentially discrete diffraction pattern, and by ‘aperiodic crystal’ we mean any crystal in which three-dimensional lattice periodicity can be considered to be absent” [9]. By this latest definition, our solid is a crystal, albeit an aperiodic one. It is a “quasiperiodic crystal” or quasicrystal for short, a word coined by Levine and Steinhardt [10].

Our surprising discovery created quite a stir and has influenced research in many fields, not just crystallography, but also materials science, physics, mathematics [11,12], biology [13,14], and even art. There have been about 10 000 papers in these fields and many conference proceedings [15]. Hundreds of quasicrystals have been found since, some with non-crystallographic axial symmetries, pentagonal, octagonal [16], decagonal [17], and dodecagonal [18]. The crystals with axial symmetries are usually periodic along the symmetry axis, and quasiperiodic in the basal plane.

Quasiperiodicity is a form of aperiodicity that has many of the attributes of periodicity. As one of their defining properties, Fourier transforms of quasiperiodic functions are discrete sets of delta-functions; they can always be expressed as a series of sine and cosine terms, but with incommensurate lengths, or a number of arithmetically independent basis vectors that exceeds the number of independent variables. Physically, a quasiperiodic object diffracts to give a pattern with sharp Bragg spots. But whereas diffraction from a periodic object forms a reciprocal lattice that can be indexed with a set of *d* reciprocal basis vectors, where *d* is the dimension, the diffraction pattern from a quasiperiodic object requires a finite number, *D* > *d*, independent basis vectors. An important consequence of this is that any quasiperiodic function can always be represented as a periodic function in *D* dimensions. The aperiodic function then is a *d*-dimensional cut of this periodic function. If *D* is infinite, the function is called almost periodic. We have so far not been concerned with almost periodicity, since in any experiment *D* is less than or equal to the number of observed reflections, and thus is finite.

As a simple example consider the one-dimensional function *f* (*x*) = cos *x* + cos *bx*. The Fourier transform consists of two delta functions. If *b* is rational, *f* is periodic, the two delta functions can be indexed with a single reciprocal lattice vector. If *b* is irrational, *f* is quasiperiodic; there are two incommensurate lengths in the Fourier transform; *D* = 2. The function *f* (*x*,*y*) = cos *x* + cos *y* is periodic in two dimensions; the quasiperiodic one-dimensional *f* (*x*) is recovered by setting *y* = *bx*. Note that there would be no diffuse scattering from a quasiperiodic object with *f* as its density function.

**Fig. 1:**
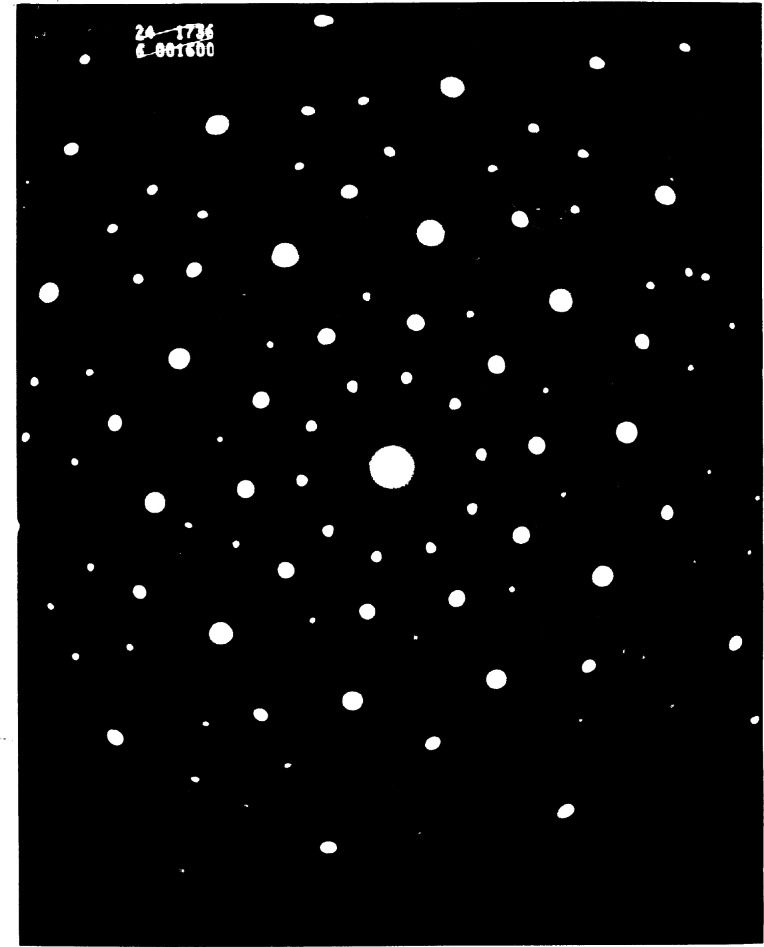
The first electron diffraction pattern from a quasicrystal [1]. Note the forbidden, 10-fold axis, the absence of systematic rows, and the need for more than three vectors to index all the spots.

Figure 1 shows the first diffraction pattern taken from a quasicrystal oriented along the 5. Note first the discrete diffraction and the apparent 10-fold symmetry. Note that there are no systematic rows; spots twice or three times as far as a bright spot are much weaker if seen at all. Note that the ratio of distances in any row is the “golden mean” $\text{τ},\left(\text{τ}=2\;\cos\;36^{0}=\left(1+\sqrt{5}\right)/2=1.618034\ldots \right)$, and that τ occurs naturally in the ratios of the magnitudes of vector sums of spots at 36^0^ from one another. Lastly note that it is impossible to index this pattern with just three reciprocal lattice vectors.

**Fig. 2:**
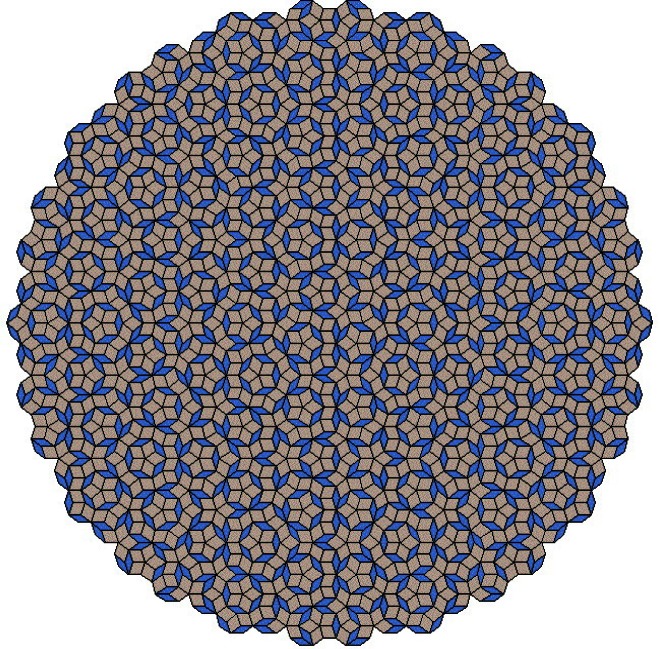
Penrose tilings are quasiperiodic. Groups of three tiles around a trivalent point look like three-dimensional cubes with 90° between line segments, but the orientation of some tiles is ambiguous. In five dimensions this ambiguity is removed, all line segments can be orthogonal, an then this entire pattern will fit between two parallel hyperplanes.

Our brains often take us to higher dimensions to simplify what is seen. Every triplet of rhombs meeting at a triple corner in the Penrose tiling in Fig. 2 can look like a three-dimensional cube, but they are arrayed in several orientations, and the same rhomb can seem to have different orientations depending on which other two neighboring rhombs it is grouped with. In five dimensions this tiling finally becomes simple and unambiguous with each edge along a specific one of five orthogonal axes and each rhomb becomes a square with a unique orientation. A zigzag path along the lines of the tiling becomes a Cartesian path in five dimensions, and a five-index coordinate system specifies each corner. In five dimensions the Penrose tiling is confined to the set of all the lattice points within a slice bounded by two parallel plane hypersurfaces with irrational orientations.

Since the (111) plane of the primitive cubic lattice is the two-dimensional hexagonal lattice, the three-dimensional hexagonal lattice can be considered as the (1110) plane of a four-dimensional cubic lattice [19]. This rational cut can simplify the understanding of indexing hexagonal structures. The 4-index specification of a point 〈hkil〉 in a four-dimensional cubic structure can be used to specify a point in the real three-dimensional hexagonal crystal. For the point 〈hkil〉 to be in the three-dimensional crystal, it has to be on the (1110) hyperplane of the four-dimensional cubic structure, i.e., it has to have *h* + *k* + *i* = 0. Distances between two such points can be computed more easily in the 4-index notation.

A physical example of a two-dimensional quasiperiodic object is the surface obtained by cutting a three-dimensional crystal by an irrational plane. In this example the three basis vectors of the periodic three-dimensional crystal are needed to describe this two-dimensional quasiperiodic surface. Because the cutting plane is irrational the surface cannot be periodic; it will never go through exactly the same point in two different unit cells. Yet when the plane comes close to the same point in some distant unit cell, another plane through that point will be very close all the way out to infinity. The aperiodic structures these planes represent will superimpose with little error all the way to infinity. That distance between the points is an approximate translation vector, whose existence depends on the specification of how small a superposition error we require. For a periodic function the superposition would be exact; the translation can be repeated indefinitely, and thus form a lattice. For a quasiperiodic function, repetition of any translation increases the mismatch, and eventually the error becomes too large; thus the translations in a quasiperiodic structure do not form a lattice, but what is called a quasilattice. But the existence of these translations is an important property of quasiperiodic functions and of quasicrystals.

There are many kinds of defects in periodic structures that have their analogs in quasiperiodic structures. Let us begin by examining how defects in a three-dimensional periodic crystal would appear on a two-dimensional aperiodic surface. Consider, for example, a metallic crystal with a CsCl ordering of a body centered cubic structure to a $Pm\bar{3}m$ space group with differing occupation of corners and body centers. Such metallic crystals commonly have internal boundaries, called antiphase boundaries, separating domains in which the site occupations are reversed. Such boundaries break the translational symmetry in an otherwise periodic crystal. Now consider a cut of such a crystal on an irrational plane. Although this cut surface is aperiodic, the domain boundary, a translation defect, would clearly be visible in the quasiperiodic surface. Dislocation lines in three dimensions, intersecting the surface, would show up in the surface as points with associated Burgers vectors. Since, apart from some small strains, the three-dimensional structure is perfect away from the dislocation, so is the quasiperiodic surface. Thus we can detect translational faults and imperfections in quasiperiodic objects. Defects in quasicrystals can be understood as defects in a higher-dimensional periodic crystal. Ordering can occur in icosahedral quasicrystals, giving rise to antiphase boundaries that are five-dimensional hypersurfaces in the six-dimensional crystal and seen as surfaces in the three-dimensional quasicrystal [20]. This boundary can also be seen in an imperfect ordering of a Penrose tiling in which adjacent corners alternate black and white. Dislocation lines in icosahedral quasicrystals arise from a four-dimensional defect surface in six dimensions. Mechanical deformation of quasicrystals is a most interesting subject. Away from the dislocation line, the quasicrystal is perfect, as it would be with dislocations in periodic crystals.

Although no new symmetry axes appear in going from two to three dimensions, higher dimensions allow new symmetries to be consistent with periodicity. For the axial groups a *n*-fold symmetry axis first becomes possible with translational symmetry if the dimensionality equals the totient of *n*, which is the number of positive integers less than or equal to *n* which are relatively prime (no common factors) to *n* [21]. This is readily illustrated for any prime number *N*, whose totient is *N*−1. Since the (11…1) hyperplane in an *N*-dimensional isometric lattice has an *N*-fold axis and the dimension of that plane is *N*−1, the rule works for all primes. Two has a totient of 1; three, four, and six have totients of 2; none have 3; five, eight, ten, and twelve have 4, etc. Thus five, eight, ten, and twelve-fold rotations first appear in four-dimensional periodicity. Icosahedral symmetry with its intersecting five-fold axes requires six dimensions. Each of the six axes in an isometric six-dimensional lattice meets the five others at right angles, giving rise to six 10-fold axes. Because there is no point group with more than one 10-fold axes in three dimensions, the cuts by irrational planes can only preserve the six 5-fold (or the six 5-fold inversion) axes of the icosahedral symmetry.

The study of quasicrystals benefited greatly from prior research in the mathematical subjects of quasiperiodic functions, aperiodic tilings, and hyperspace crystallography. The latter had already been applied in the study of modulated crystals [22]. Modulated structures had been found long before the discovery of quasicrystals and had provided some well-documented and understood exceptions to periodic crystals. Because they could be considered as small incommensurate distortions of periodic structures with a crystallographic point group, they could be fit into the schemes of crystallography. But the incorporation of the modulation wavelength as an additional length provided an impetus for the development of hyperspace crystallography which allowed a periodic indexing in the higher dimension. Modulated structures could then be treated as cuts on irrational planes, and sometimes as projections of a slice, of a four or higher dimensional periodic structure. In an ideal modulated structure, each spot, including the satellites, is a Bragg peak, indexed with more than three, usually four, numbers.

Consider an icosahedral structure to be an irrational cut of a six-dimensional cubic structure with a single lattice parameter. Indexing requires six numbers, which is obvious in six dimensions, but is also true for three. In a three-dimensional indexing using three orthogonal axes in a Cartesian system, two indexes are required along each axis, and six number specify each spot, (*h* + *h′τ*, *k* + *k′τ*, *1* + *1′τ*) [23]. Indexing of icosahedral powder patterns is also straightforward; the ambiguities resulting from superpositions (such as (330) and (411) in bcc powder patterns) are infrequent. After a lattice parameter has been selected, indexing for all six numbers for single crystals is unambiguous in either three or six dimensions. Using synchrotron radiation from a single AlCuFe quasicrystal, Moss and coworkers have measured intensities of about 1200 crystallographically distinct peaks, every peak found using a single icosahedral (quasi)lattice parameter and a six-parameter icosahedral indexing [24].

Structure determinations would seem like a hopeless task. Has one to describe the structure of an aperiodic solid out to infinity? Because there is periodicity in the higher dimensions, one needs only to describe the content of one unit cell (hypercell) in the higher dimensional space. Structure determination in six dimensions is not very different from what it is in three. Once the diffraction peaks from single crystals (or lines from powders) have been indexed in six dimensions, or in three with six basis vectors, and their corrected intensities measured, the diffraction pattern can be considered either on a three-dimensional reciprocal quasilattice or on a six-dimensional periodic lattice. They are completely equivalent to another, but standard methods of crystallographic structure determination for periodic structures are applicable with little modification to the six-dimensional data.

Indexing allows Patterson functions to be directly obtainable in three or six dimensions from powder data. They have the directional information lost in a radial distribution function. Although the three-dimensional Patterson functions are aperiodic and complicated with many peaks, near the origin they bear striking similarities to Patterson functions of related periodic approximants, large cell periodic crystals with compositions slightly different from quasicrystals [25]. Thus the local atom packing of quasicrystals are found to be very similar to that of corresponding periodic phases. Patterson functions in six dimensions are usually found to be much simpler, with only a few peaks in each unit cell. Actual structure determinations have now been carried out for several quasicrystals with very good residuals [26,27]. Atom positions are described in the six-dimensional unit cell by three-dimensional surfaces; the intersections of these surfaces periodically repeated in six dimensions by the irrational three-dimensional plane are the points that describe the atom positions in the three-dimensions of quasiperiodic structures. In analogy with the finding of three dimensional periodic structures by fitting balls with atomic radii together, plausible quasiperiodic structures have been constructed by fitting atomic surfaces into six dimensional unit cells [28]. Another technique exploits the known structures of periodic approximants to convert the structure determination of the related quasicrystals to the standard crystallographic structure refinement problem [29].

Periodic crystals can be considered a tiling of unit cells, each decorated with atoms. Tilings with noncrystallographic symmetries occur in art where the mathematicians’ rules about having a limited number of congruent tiles and leaving no gaps need not be met. The discovery by mathematicians of aperiodic tilings preceded that of quasicrystals. Penrose’s tilings with 5-fold symmetry seem particularly pertinent; they are quasiperiodic and their diffraction pattern is strikingly similar to that of the 5-fold zone of icosahedral quasicrystals [30]. By analogy some of the early models were based on atomic decorations of three-dimensional versions of Penrose tiles as if each of the different tiles had the same decoration of filled atom positions.

Three-dimensional structures that give sharp diffraction are either periodic, if the indexing requires three basis vectors, quasiperiodic, if the indexing requires a finite number, more than three, and almost periodic, if the indexing requires an infinite number. All the mathematical interest had been with almost periodicity; any quasiperiodic structure is periodic in a higher dimension.

There was considerable initial resistance to quasicrystals. My own initial reaction was that we were seeing a quintuple twin, often seen in cubic crystal, but that was easily ruled out with data presented in our paper. The angles between the (111) twinning planes (arccos (1/3) ≈ 70.53°) in cubic crystals are sufficiently close to 72° that five wedge shaped periodic cubic crystal can fill space with some easily detected strain or extra material to fill the missing 7.36°. Twins lead to a superpositioning of five reciprocal lattices, each giving systematic rows of periodically spaced diffraction spots. The absence of such systematic rows argues against twinning. The other possibility was a very large unit cell with a structure that will give the strange extinctions to conform to the lack of systematic rows of spots that we now know is a characteristic of diffraction from quasicrystals. Assuming we had a periodic low-symmetry crystal, we searched unsuccessfully to fit the data with cell constants up 2.5 nm. Even though either the twinning or the large unit cell were plausible alternate explanations, Linus Pauling became one of the vocal opponents by proposing a double-kill, both a large unit cell and what he called icosatwinning. His initial structure, based on his often successful method of fitting atoms together, had a face centered cubic unit cell with a lattice constant of 2.67 nm, containing 1168 atoms (292 atoms per primitive cell). His claim [31] to fit our powder data led him to write that there was only 1 chance in 10 000 that this unit cell could be wrong, but he ignored that his indexing could not fit our published single crystal pattern. A few years later, he found it necessary to propose another cell, this time a primitive cubic structure with a lattice constant of 2.34 nm, containing 820 atoms [32]. Either of his structures would qualify as an approximant, but to the best of my knowledge, no one has yet reported finding either of them.

Quasicrystals provided win-win opportunities for crystallographers: If we were mistaken about them, expert crystallographers could debunk us; if we were right, here was an opportunity to be a trail blazer. While many crystallographers worldwide availed themselves of the opportunity, U.S. crystallographers avoided it, to a large extent because of Pauling’s influence. The demonstration by E. Prince that tilings with five-fold symmetry would give discrete diffraction pattern was a notable exception [33].

The systems that form quasicrystals additionally often give periodic crystals with large unit cells, called periodic approximants; sometimes there is even a sequence of approximants with ever larger cell constants [34]. The Frank-Kasper phases [35] turned out to be examples of periodic approximants to quasicrystals that were found later. Because their diffraction spots are periodically arrayed, approximants are easily distinguished from quasicrystals. Quintuple twinning of approximants is sometimes seen, as is one case of triple twinning of a quasicrystal, giving an apparent 30-fold diffraction pattern [36].

Much has been written about why quasicrystals exist. Although it could not be proven, it was taken as plausible by many eminent scholars that the lowest energy configuration of a set of identical atoms or molecules would be periodic. Similarly it was assumed that the lowest energy configuration of any mixture of atoms or molecules would be a periodic arrangement of identical unit cells forming a stoichiometric compound, or a mixture of such periodic structures. Radin has shown quite the opposite; for almost any assumed interaction between molecular units, the lowest energy is a quasiperiodic rather than a periodic structure [37]. He has raised the question about whether periodic crystals exist because kinetics are too slow to reach the lowest energy state, or whether there is something special about the interactions obtained from quantum mechanics.

To this date all quasicrystals have been metallic. In metallic structures interatomic distances are determined, but bond angles do not seem to matter. Local atomic configurations thus obtained often do not pack well into periodic structures. Even the simplest one-component metallic structures seem to favor regular tetrahedral arrangements that do not fill space. What other local configuration is needed to fill the gaps, and does that lead to the orientational order seen in quasicrystals and periodicity or quasiperiodicity? Structures are determined by a trade-off between low energy local packing and the occasional higher energy configuration that is geometrically necessary. In order to have a periodic space-filling arrangement, both the face-centered and hexagonal close packed structures, for example, introduce the octahedra, a configuration which one expects to have a higher energy. The stable quasicrystals and the approximants are made of two [38] or more chemical components, allowing irregular tetrahedra that have a better chance of filling space. Whether the adjustments happen to lead to a periodic approximant or to a quasicrystal often seems to hinge on small changes in composition or temperature.

While periodic and quasiperiodic structures always give discrete diffraction, what other kinds of aperiodic structures diffract [39]? Mathematicians have found a veritable “zoo” of orderly dispositions of points in space [11]. Have any of the many that are not quasiperiodic been found in nature or made in the laboratory? An isotropic solid structure was found at NIST in a four-component system in which quasicrystals exist at somewhat different compositions. But while metallic glasses usually result from any remaining melt that is cooled too rapidly to crystallize, this solid grew first as if it were a crystal, with an interface and at a composition different from the melt [40]. On continued cooling the melt crystallized around this solid. Is this a physical realization of one of the many other orderly, arrangements of atoms, discussed by mathematicians that is not quasiperiodic?

Lattices are considered important factors in many physical problems. For a long time a three-dimensional coincidence site lattice was deemed so important that laws of twinning were based on the existence of a periodic arrangement of a fraction *S* of lattice sites of both twins, even if the lattice sites are not occupied by atoms. Coincidence sites are still considered important in the theory of grain boundaries, except that the twinner’s *S* has become a *Σ*. But why should such a three-dimensional lattice be so important? The energy surely depends only on the fit of atoms at the twin interface or grain boundary. An extreme case is a merohedral twin for which *S* = 1 in which the lattice is continuous through the twin boundary. These twins occur in cases where the motif has less symmetry than the lattice; the twin is formed when the motif is rotated by a symmetry operation of the lattice but not of the motif. We found the opposite case in an arrangement of several approximant crystals [41]. Here the icosahedral motif has approximate symmetry operations that are not present in the cubic lattice. Quasitwins occur when the lattice rotates by 72° about an irrational 〈1*τ* 0〉 axis while the motif retains its orientation across the grain boundary. The motif has long range orientational order across the boundary as it does in quasicrystals, surely for energy reasons.
